# Ruxolitinib rechallenge in combination with hydroxyurea is effective in reverting cachexia and reducing blood transfusion demand and splenomegaly symptoms in a patient with primary myelofibrosis

**DOI:** 10.1007/s00277-017-2938-5

**Published:** 2017-02-14

**Authors:** Claudio Cerchione, Ilaria Peluso, Davide Nappi, Anna Emanuele Pareto, Marco Picardi, Vincenzo Martinelli, Fabrizio Pane

**Affiliations:** Hematology Azienda Ospedaliera Universitaria Federico II, Via Pansini 5, 80131 Naples, Italy

**Keywords:** Primary myelofibrosis, Cachexia, Ruxolitinib, Hydroxyurea, Splenomegaly

Dear Editor,

Myelofibrosis (MF) is a myeloproliferative neoplasm (MPN) whose pathogenesis mainly involves JAK/STAT signaling; approximately 65% of patients carry V617F-JAK2 mutation with a gain-of-function mechanism [1, 2]. Hydroxyurea is recommended as the first-line therapy for MF in low and intermediate-1 patients, whereas ruxolitinib, an orally available and selective JAK2-inhibitor, is recommended in International Prognostic Scoring System (IPSS) intermediate-2 and high-risk patients as front-line treatment of symptoms and splenomegaly in non-transplant candidates [3].

We describe the case of a 57-year-old Caucasian man with primary MF (PMF). The patient was 166 cm in height and weighed 60 kg; before diagnosis, he had been in fair physical condition. At diagnosis (November 2012), his main symptoms were early satiety and a sense of fullness in the left upper abdomen that rapidly deteriorated into cachexia. His blood count showed anemia and leukocytosis, and a physical examination revealed splenomegaly (10 cm from costal margin); size measured by ultrasound scan [4] was 22 cm (longitudinal diameter) × 14 cm (transverse diameter) with a spleen volume of 2700 mL. Bone marrow biopsy demonstrated grade 3 fibrosis (MF = 3) and the presence of *JAK2*-V617F mutation. In December 2012, cytoreductive therapy with hydroxyurea (1 and 2 capsules daily on alternate days) was started, obtaining a stable disease for few months. After 3 months, systemic symptoms and splenomegaly worsened. The patient was cachectic, his weight had fallen to 47 kg, and his spleen was of hard consistency and had enlarged, extending to the iliac fossa. He had lack of appetite, he was having difficulty eating, and his quality of life had deteriorated badly. The severity of the patient’s condition led to the consideration of other treatment options.

**Fig. 1:**
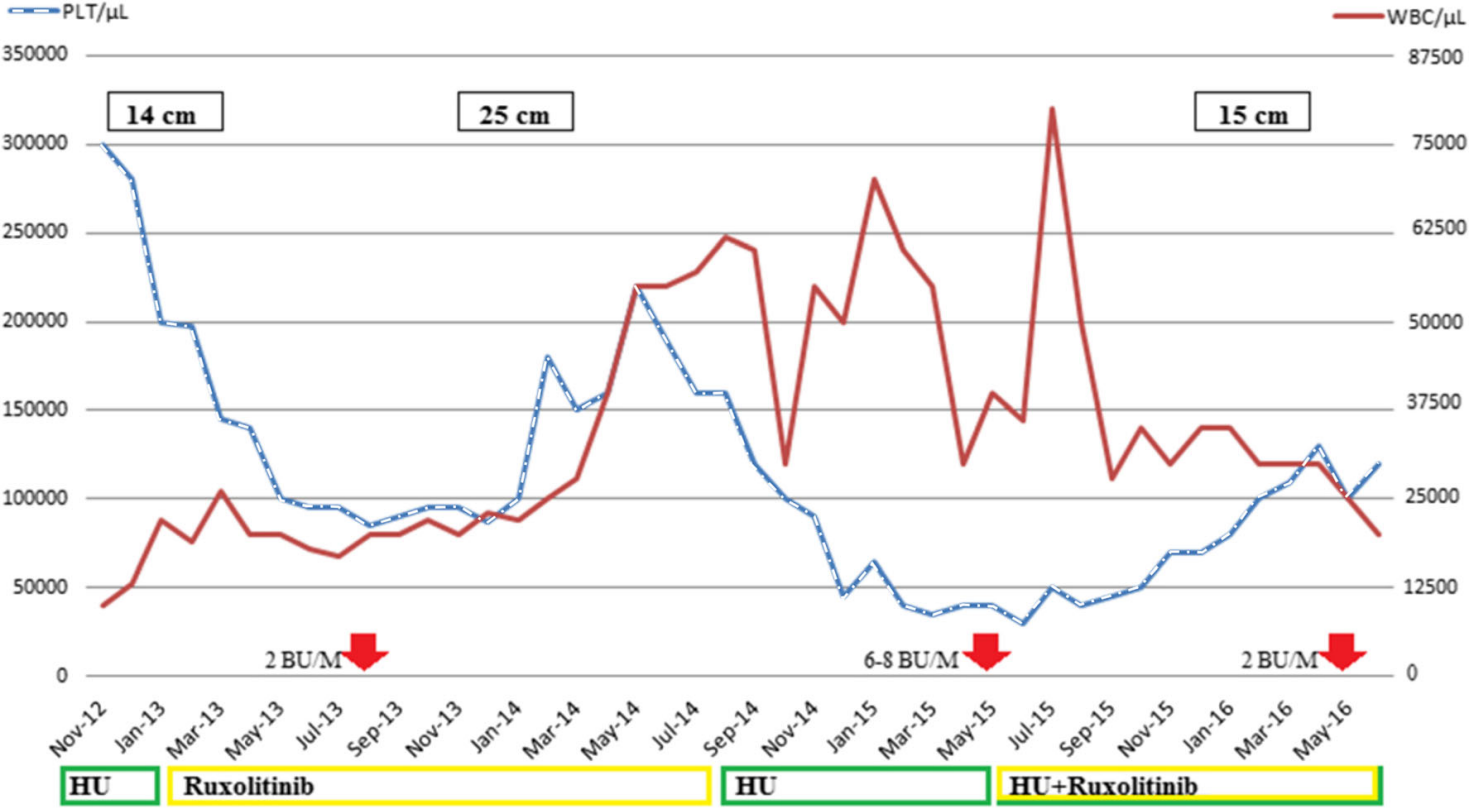
Patient clinical history showing platelet (PLT) count (*dashed line*), white blood cell (WBC) count (*solid line*), blood units per month (BU/M; *broad arrows*), spleen longitudinal diameter (*black rectangles*), and timeline of hydroxyurea (HU) and ruxolitinib administrations

The patient refused allogeneic stem cell transplantation after becoming aware of transplant-related risks and peri-transplant mortality. Therefore, treatment with ruxolitinib was initiated, initially at 10 mg twice daily (bid), and reduced to 5 mg bid in response to grade 3 thrombocytopenia. The patient experienced only partial relief from symptoms and, in September 2014, ruxolitinib was discontinued due to severe leukocytosis and very poor compliance. After hydroxyurea was reintroduced to control leukocytosis, there was a considerable increase in the need for blood transfusions over subsequent months (up to 8 units/month; Fig. 1) and spleen size increased, reaching 27.8 cm longitudinal diameter. Following poor compliance with gastroprotective drugs, the patient required hospitalization in April 2015 for gastric bleeding, and hydroxyurea therapy was stopped due to severe anemia and thrombocytopenia (30,000/mm^3^).

Hydroxyurea was reintroduced with palliative intent after 1 month and, in June 2015, low-dose ruxolitinib (5 mg bid) rechallenge was undertaken, in combination with hydroxyurea (1 and 2 capsules daily on alternate days for 5 days/week). The ruxolitinib dose was increased to 10 mg bid after 1 month, and after 2 months (September 2015), the patient experienced a dramatic reduction in spleen size with substantial relief from symptomatic splenomegaly (longitudinal diameter 15.6 cm), control of anemia and leukocytosis, and improved nutritional status, with an increase in appetite and an increase in weight to 55 kg. He regained a decent quality of life and was able to resume routine activities, such as shopping.

Resolution of cachexia and substantial improvement in clinical status continued and, as of May 2016, the patient was continuing combination treatment with ruxolitinib (10 mg bid) and hydroxyurea.

Single-agent ruxolitinib is effective in improving splenomegaly, systemic symptoms, and overall survival, compared with placebo and standard treatment, in patients with intermediate-2 or high-risk MF [5, 6]. As with hydroxyurea, significant anemia and thrombocytopenia are the most common side effects, often requiring discontinuation [7]. However, despite the combination of ruxolitinib with a cytoreductive agent, we obtained control of leukocytosis and anemia with concurrent increase in platelet count to stable normal values, without the expected synergic cytotoxic effects. Of interest, the safe and effective use of combination of ruxolitinib plus hydroxyurea in reducing platelet count and splenomegaly in a patient with uncontrolled thrombocytosis on ruxolitinib monotherapy has been described [8]. Furthermore, in addition to its primary anti-myeloproliferative action via JAK2 inhibition, ruxolitinib appears to exert a remarkable improvement in cachexia status [9], as observed in our case.

In conclusion, combined ruxolitinib plus hydroxyurea effectively controlled myeloproliferation without worsening anemia, instead leading to a remarkable decrease in the need for blood transfusions. Our patient’s cachectic status was reverted, and overall quality of life dramatically improved.
